# Aromatic Medicinal Plants of the Lamiaceae Family from Uzbekistan: Ethnopharmacology, Essential Oils Composition, and Biological Activities

**DOI:** 10.3390/medicines4010008

**Published:** 2017-02-10

**Authors:** Nilufar Z. Mamadalieva, Davlat Kh. Akramov, Elisa Ovidi, Antonio Tiezzi, Lutfun Nahar, Shahnoz S. Azimova, Satyajit D. Sarker

**Affiliations:** 1 Laboratory of Chemistry of Glycosides, Institute of the Chemistry of Plant Substances AS RUz, Tashkent 100170, Uzbekistan; a.davlat@inbox.ru (D.K.A.); genlab_icps@yahoo.com (S.S.A.); 2 Department for the Innovation in Biological, Agro-food and Forestal Systems, Tuscia University, Viterbo 01100, Italy; eovidi@unitus.it (E.O.); antoniot@unitus.it (A.T.); 3 School of Pharmacy and Biomolecular Sciences, Faculty of Science, Liverpool John Moores University, Liverpool L3 3AF, UK; L.Nahar@ljmu.ac.uk (L.N.); S.Sarker@ljmu.ac.uk (S.D.S.)

**Keywords:** Uzbekistan, Lamiaceae, traditional use, aromatic plants, essential oils

## Abstract

Plants of the Lamiaceae family are important ornamental, medicinal, and aromatic plants, many of which produce essential oils that are used in traditional and modern medicine, and in the food, cosmetics, and pharmaceutical industry. Various species of the genera *Hyssopus*, *Leonurus*, *Mentha*, *Nepeta*, *Origanum*, *Perovskia*, *Phlomis*, *Salvia*, *Scutellaria,* and *Ziziphora* are widespread throughout the world, are the most popular plants in Uzbek traditional remedies, and are often used for the treatment of wounds, gastritis, infections, dermatitis, bronchitis, and inflammation. Extensive studies of the chemical components of these plants have led to the identification of many compounds, as well as essentials oils, with medicinal and other commercial values. The purpose of this review is to provide a critical overview of the literature surrounding the traditional uses, ethnopharmacology, biological activities, and essential oils composition of aromatic plants of the family Lamiaceae, from the Uzbek flora.

## 1. Introduction

The Republic of Uzbekistan is located in the center of Eurasia. About 85% of its territories are deserts, and about 15% are mountains and foothills. The Uzbek flora accounts for approximately 4350 species of vascular plants, including large numbers of endemic, endangered, and globally important species. Plants endemic to Uzbekistan constitute 20% of all plants; and a majority of these grow in the mountains. The floristic data for several regions of Uzbekistan is imperfect, and studies are continuing [1,2].

One of the famous medicinal aromatic plant families is the Lamiaceae family (alt. Labiatae), also known as the mint family. Aromatic medicinal plants from this family have long been used in Uzbek traditional medicine. The aim of this review is to present a critical overview of the ethnopharmacology, ethnobotany, phytochemistry, essential oils composition, and biological activities of medicinal plants of the family Lamiaceae, e.g., *Hyssopus seravschanicus*, *Leonurus panzerioides*, *L. turkestanicus*, *Mentha longifolia* var. *asiatica*, *Nepeta alatavica*, *N. olgae*, *Origanum tyttanthum*, *Perovskia scrophulariifolia*, *Phlomis thapsoides*, *Salvia korolkovii*, *S. sclarea*, *Scutellaria immaculata*, *S. ramosissima*, *S. schachristanica*, *Ziziphora clinopodioides*, and *Z. pedicellata*, from the Uzbek flora. This review has been compiled using references from major databases, such as Science Direct, SciFinder, Pubmed, and Google Scholar Databases. The search included articles published to date.

## 2. Essential Oils from the Uzbek Lamiaceae Species

### 2.1. Hyssopus seravschanicus (Dubj.) Pazij

**Figure 1:**
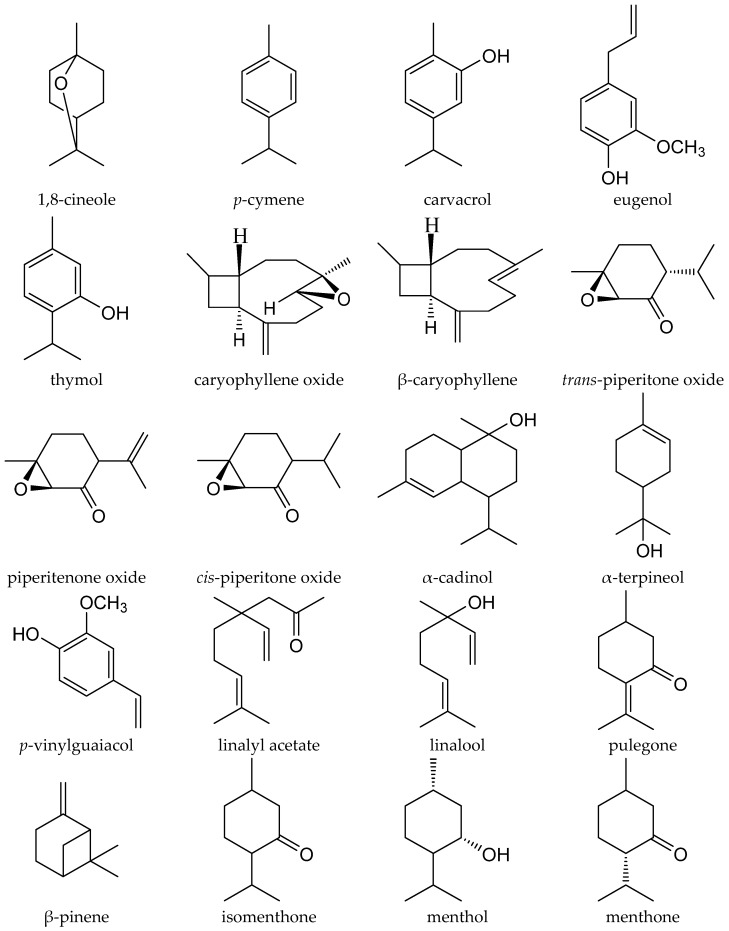

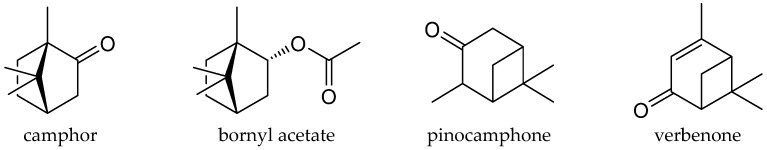
Major compounds of the essential oils of the Lamiaceae family.

The genus *Hyssopus* L. consists of over 15 species worldwide. *Hyssopus* is a source of volatile oils and its constituents are mostly sesquiterpenes, bicyclic monoterpenes, and some acids [3]. Only one species of this genus, *Hyssopus seravschanicus*, grows in Uzbekistan [2]. *Hyssopus seravschanicus* Pazij is a perennial, branched, semi-shrub that is native to the the Republic of Uzbekistan. Several researchers have studied the essential oils content of *H. seravschanicus* [3,4,5]. The most abundant compounds identified in the essential oils of *H. seravschanicus*, were pinocamphone (71.0%), β-pinene (8.6%), 1,8-cineole (6.4%), carvacrol (1.6%), *cis*-ocimene (1.4%), *p*-cymene (1.3%) and sabinene (1.3%) (Figure 1) (Table 1) [5].

### 2.2. Leonurus panzerioides Popov

The genus *Leonurus* L. (subfamily Lamioideae) comprises 25 species [6]. The *Leonurus* are characterized by the presence of iridoid glycosides and a lower content of essential oils. This genus is represented in Uzbekistan by four species. *Leonurus panzerioides* Popov is a perennial shrub that grows in Western Tien Shan and the Pamir-Alay mountains, on stony and gravelly slopes [2,7]. A tincture of the herb of *L. panzerioides* has been known to possess a sedative effect, which is twice as strong as the effect of a valerian tincture (Table 1) [8]. The main constituents of the essential oils of *L. panzerioides* were found to be eugenol (30.93%), *p*-vinyl guaiacol (15.77%), dihydroactinidiolide (8.95%), phenyl ethyl alcohol (6.51%), verbenone (5.83%), and *p*-cymen-8-ol (5.24%). Twenty-four compounds were identified in the oil of *L. panzerioides*, which accounted for 99.98% of the total oil [9].

### 2.3. Leonurus turkestanicus V. I. Krecz. & Kuprian

*Leonurus turkestanicus* V. I. Krecz & Kuprian is a perennial shrub that grows in the plains and highlands of Asia on stony, shallow-soiled slopes, floodplains, streamsides, and among trees and other shrubs [10]. A decoction of the above-ground parts is used to treat various ailments of the heart, stomach, and nervous system [8,11]. Previous phytochemical investigations of the aerial parts of *L. turkestanicus* identified flavonoids, iridoids, alkaloids, and fatty acids. Thirty-nine chemical constituents were detected by GC–MS analysis of the essential oils of *L. turkestanicus*, representing 99.98% of total oil components. The essential oil from the aerial parts of *L. turkestanicus* had oxygenated monoterpenoids and sesquiterpenoids as the major components, and thus shared the characteristics of thymol chemotype. The principal constituents of the essential oils of this species were found to be thymol (40.10%), octen-3-ol (13.07%), carvacrol (5.83%), and β-caryophyllene (5.61%) (Table 1) [9].

### 2.4. Mentha longifolia var. asiatica (Boriss) Rech. f.

*Mentha* L. (mint) is a well-known genus due to its medicinal and aromatic value. It is represented by about 19 species and 13 natural hybrids, mainly perennial herbs, which grow wildly in damp or wet places throughout the temperate regions of Europe, Asia, Africa, Australia, and North America. Species of the genus *Mentha* have been reported to contain a range of components, including cinnamic acids, flavonoids, and steroidal glycosides. However, the main active component of the genus *Mentha* is essential oil, which is reported to govern its various properties [12]. Three species of the *Mentha* grow in Uzbekistan [2]. *Mentha longifolia* var. *asiatica* (Boriss) Rech. f., is commonly used as a cooking herb by the people. It has a pleasant taste and is a popular flavouring for food and drink. Thirty-seven compounds were characterized from *M. longifolia* var. *asiatica*, representing 97% of the total components detected. The major constituents of the oil were found to be *trans*-piperitone oxide (64.51%), piperitenone oxide (12.34%), *cis*-piperitone oxide (7.24%), thymol (2.60%), and spathulenol (2.36%) (Table 1) [13].

### 2.5. Nepeta alatavica Lipsky

The genus *Nepeta* L. comprises perennial or annual herbaceous, small shrubs, and rarely includes trees. It is comprised of more than 200 species. This genus has a widespread distribution in the temperate regions of Asia, Europe, and North Africa. Some *Nepeta* species are widely used in traditional medicine, due to their diuretic, antispasmodic, anti-asthmatic, febrifuge, emmenagogue, sedative, and antiseptic properties [14]. In Uzbekistan, the genus *Nepeta* is represented by 19 species [2]. *Nepeta alatavica* Lipsky is a perennial plant that grows in the Tien Shan mountains (Kyrgyz Alatau ridges, Talas Alatau, Karzhantau, Ugam, Pskem, Chatkal mountains). The major constituents of the essential oils of *N. alatavica* were thymol (48.5%), carvacrol (7.5%), verbenone (7.7%), and 1-octen-3-ol (4.1%) (Table 1) [15].

### 2.6. Nepeta Olgae Regel

In Uzbekistan, *Nepeta olgae* Regel grows as an aromatic perennial plant in the foothills and lowlands of the Syrdaya region, Kyzylkum, and the Surkhan-Sherabad and Ferghana Valleys [2]. The leaves of this plant have the richest odour intensity, and are used primarily for its fragrance. The *N. olgae* oil is dominated by acetylcyclohexene (31.5%), 4-tridecyne (13.2%), 2-methyl cyclopentanone (6.8%), and 1,8-cineole (6.0%) (Table 1) [15].

### 2.7. Origanum tyttanthum Gontsch

The genus *Origanum* L. consists of 43 species and 18 hybrids; most of which are distributed through the eastern Mediterranean region [16]. Only one species, *Origanum tyttanthum* Gontsch., is found in Uzbekistan. This herbaceous perennial, rhizomatous plant, grows on rocky and pebbly slopes. The plant contains phenolic glycosides, lipids, and coumarins [17]. Forty compounds were characterized, representing 98.6% of the total components in its essential oils [18]. The major components of the essential oils were reported to be carvacrol (42.76%), thymol (27.18%), γ-terpinene (9.50%), *p*-cymene (5.90%), and *β*-bisabolene (2.65%) [18]. In a different study [19], *O. tytthanthum* oils were analyzed to reveal about 30 compounds, 20 of which were identified, and the major ones were thymol and carvacrol (48%–89%). The above-ground part of *O. tyttanthum* was found to contain 0.3%–2.1% oil. The oil content in the different parts of this plant changes considerably, depending on the different conditions: phase of vegetation, sunlight exposition, and altitude of plant growth (Table 1).

### 2.8. Perovskia scrophulariifolia Bunge

The genus *Perovskia* Kar. is made up of seven different species [20], four of which grow in Uzbekistan [2]. *Perovskia scrophulariifolia* Bunge is an aromatic sub-shrub featuring extremely branched stems, paniculate leaves, and small flowers. It is mainly known for its ornamental and flavouring qualities. From the aerial parts of *P. scrophulariifolia*, abietane-type diterpenoids, flavone glycosides, anthocyanins, and coumarins were isolated and identified [21]. Constituents of the essential oil of the aerial parts of *P. scrophulariifolia* growing in Turkmenistan, were reported as being borneol, camphene, geraniol, linalool, α-pinene, sabinene, terpinene, and terpinolene, without quantitative indication [22]. In another study, the essential oil of *P. scrophulariifolia* [23] revealed the presence of a total of at least 20 components. Farnesene and the products of its oxidation, and some modifications of sesquiterpene hydrocarbons with a naphthalene nucleus, were identified. Of the monoterpenoids, bornyl acetate predominated, and of the sesquiterpenoids, α- and β-caryophyllenes were identified. In a separate study, major constituents found in the oils *P. scrophulariifolia* were as follows: 1,8-cineole (11.0%), caryophyllene oxide (10.0%), camphor (9.0%), humulene epoxide II (7.9%), bornyl acetate (7.8%), and *p*-cymene (5.7%) (Table 1) [22].

### 2.9. Phlomis thapsoides Bunge

The genus *Phlomis* L. consists of 75 species of perennial shrubs, occurring from the Mediterranean to Central Asia. In Uzbekistan, the genus *Phlomis* is represented by 15 species. *Phlomis thapsoides* Bunge is a perennial herb growing wild in western Pamir-Alay. The aerial parts of this species are used in some areas of Asia for feeding animals, and to dye wool and silk [2]. Previous phytochemical studies of the aerial parts of *P. thapsoides* afforded iridoids, sterols, aliphatic ketones, and essential oils [24]. The GLC-MS analysis of the essential oil obtained from the aerial parts of *P. thapsoides*, identified phenylethyl alcohol (6.81%), *trans*-3-hexenol (5.55%), 1-octen-3-ol (5.10%), α-cadinol (4.92%), α-muurolol (4.67%), and linalool (3.69%) as the main volatile constituents (Table 1).

### 2.10. Salvia korolkovii Regel et Schmalh. (syn. Arischrada korolkovii)

The *Salvia* L. (sage) belongs to the Mentheae Tribe, and is the largest and most diverse genus of the Lamiaceae. This genus contains over 900 species, throughout the world. Many species of the *Salvia* have been used worldwide as a flavouring agent, as well as in traditional herbal medicine. A total of 21 species of the *Salvia*, including *Salvia korolkovii* Regel et Schmalh., are native to Uzbekistan [2,25]. This plant is endemic to Uzbekistan, and one of the most important aromatic plants and sources of essential oils. The main constituents of the essential oils of *S. korolkovii* were found to be 1,8-cineole (29.3%), camphor (9.8%), β-caryophyllene (8.5%), bornyl acetate (7.7%), caryophyllene oxide (7.2%), borneol (5.6%), camphene (3.4%), and limonene (3.3%) (Table 1) [26].

### 2.11. Salvia sclarea L.

*Salvia sclarea* L. (clary sage) is a well known aromatic plant, from which an oil used to be produced in large quantities in the former Soviet Union. Apart from the various medicinal uses, the essential oils of clary sage are widely applied in the food and cosmetic industries, wine making. and as a tobacco flavouring agent [27]. In addition, some authors indicated that the oil composition of *S. sclarea* was affected by the method of isolation, as well as by the plant origins and organs used for oil isolation [28,29,30]. The principal components of the oils of *S. sclarea* collected from Uzbekistan were reported to be linalool (22%–32%), linalyl acetate (25%–51%) α-terpineol (10.0%–11.0%), and geranylacetate (5.4%–6.7%) (Table 1) [27].

### 2.12. Scutellaria immaculata Nevski ex Juz

The genus *Scutellaria* L. includes about 350 species, commonly known as skullcaps [31], 38 species of which grow in Uzbekistan [2]. *Scutellaria immaculata* Nevski ex Juz. is a semi-shrub featuring beautiful white flowers, and grows on rocky and gravelly slopes. The flavonoids of this species have been well studied [32]. Constituents of the essential oil of the aerial parts of *S. immaculata* growing in Uzbekistan were reported as including acetophenone (30.39%), eugenol (20.61%), thymol (10.04%), linalool (6.92%), 1-octen-3-ol (2.89%), 4-vinylguaicol (2.50%), and 1,8-cineol (2.25%) (Table 1) [33].

### 2.13. Scutellaria ramosissima Popov

*Scutellaria ramosissima* Popov is native to Uzbekistan and grows in Northern Tien Shan, Pamir-Altay mountains (Central Asia), on the midlands of the high-altitude belt. In Uzbek folk medicine, water extracts (tea and infusion) of *S. ramosissima* are widely applied for epilepsy, inflammation, allergies, and nervous tension. The bioactivity of the plant is most likely due to the major components: flavonoids. The chloroform extract of *S. ramosissima* showed potent cytotoxic effects to *Trypanosoma brucei* TC221, and HeLa, HepG-2, and MCF-7 cancer cells [32]. The essential oils from *S. ramosissima* showed that germacrene D (23.96%), β-caryophyllene (11.09%), linalool (9.63%), hexadecanoic acid (8.34%), caryophyllene oxide (5.90%), eugenol (5.29%), acetophenone (4.67%), thymol (3.01%), and 4-vinylguaicol (2.42%), were the principal components (Table 1) [33].

### 2.14. Scutellaria schachristanica Juz

*Scutellaria schachristanica* Juz is a perannial that grows in the rocky and gravelly slopes of the highlands of Pamir-Alai (ridges Turkestan and Zeravshan). Several flavonoids were isolated from the aerial parts of this plant. The methanol/water extracts of *Scutellaria* are known as potent-free radical scavengers [32]. However, the essential oils obtained from the aerial parts of *S. schachristanica* exhibited weaker antioxidant effects in the DPPH, ABTS, and FRAP assays. The oil of the Uzbek *S. schachristanica* was reported to be largely composed of acetophenone (34.74%), linalool (26.98%), eugenol (20.67%), 1-octen-3-ol (3.73%), β-terpineol (3.57%), 2-methoxy-*p*-cresol (1.89%), and 4-vinylguaicol (1.64%) (Table 1) [33].

### 2.15. Ziziphora clinopodioides Lam. (syn. Ziziphora brevicalyx Juz)

The species of the genus *Ziziphora* are annuals or perennials, and herbaceous or sub-shrubs. The world population of this genus is represented by more than 30 different species. The *Ziziphora* species are rich in flavonoids, caffeic acid derivatives, fatty acids, triterpenes, and sterols. The essential oils of *Ziziphora* have been well studied [34]. In the flora of Uzbekistan, this genus consists of eight species, including *Ziziphora capitata* L., *Z. clinopodioides* Lam., *Z. interrupta* Juz., *Z. pamiroalaica* Juz., *Z. pedicellata* Pazij & Vved., *Z. persica* Bunge, *Z. suffruticosa* Pazij & Vved., and *Z. tenuior* L. [2].

*Ziziphora clinopodioides* Lam. is an important aromatic, edible medicinal plant. The leaves, flowers, and stem of the plant are frequently used as a wild vegetable, or as an additive to foods. The chemical constitutions of *Z. clinopodioides* growing in Iran, Turkey, Kazakhstan, Altai Republic (Russia), Tajikistan, and Urumqi (China), have been previously analyzed [35]. The compositions of the samples of the essential oils from the inflorescences and leaves collected in Uzbekistan, were determined [36]. The main component of the essential oils of the inflorescences and leaves was identified as pulegone, a substance that is characteristic of the species which were previously investigated. The amount of pulegone in the inflorescences was 88%, and was 75% in the leaves ((in leaves: pulegone (75.0%), menthone (9.6%), menthol (4.7%), caryophyllene (1.5%), pinocamphone (1.5%), limonene (1.4%); in inflorescences: pulegone (88.0%), menthone (3.2%), limonene (1.6%), pinocamphone (1.1%), caryophyllene (0.9%), menthol (0.5%)). The essential oil of the inflorescences differed from that of the leaves, by the presence of α-pinene, sabinene, and myrcene, and also by the absence of substances such as linalool, linalyl acetate, carvacrol, and α-cedrene, that were present in the essential oil of the leaves.

### 2.16. Ziziphora pedicellata Pazij et Vved

*Ziziphora pedicellata* Pazij Vved. grows on the stony, gravelly slopes of the foothills and midlands of Tien Shan. This plant contains organic acids, essential oil, vitamin C, saponins, and flavonoids. The seeds and leaves contain carotenoids and lipids. The flowers contain terpenes [8,37]. The essential oils obtained by hydro-distillation were analyzed, and a total of 31 compounds were identified from *Z. pedicellata*. The essential oils of *Z. pedicellata* were rich in the oxygenated monoterpenes pulegone (62.0%), isomenthone (11.5%), menthol (9.2%), menthone (5.5%), and β-pinene (1.0%) (Table 1) [38].

## 3. Ethnopharmacology and Biological Activities of Uzbek Lamiaceae Family Plants

Plant species from the Lamiaceae family have been used in herbal medicine for thousands of years. Traditional applications of the Lamiaceae family show high applicability as a common tea, flavours, insect repellant, in flu control, and as an anti-inflammatory, sedative, and analgesic. Mainly essential oils, terpenoids, phenolic compounds, flavonoides, and iridoids, have been reported from the members of this family. Many of the medicinal uses are presumed to be connected to the terpenic constituents of the essential oils of these plants.

Table 1 presents the various ethnopharmacological uses of the Lamiacea species, that have been widely used in Uzbek traditional medicine. The local names, as well as the collection location, growing environment, and their essential oil composition, are also included.

## 4. Conclusions

The present review provides, for the first time, an updated compilation of the documented ethnopharmacological information in relation to the ethnomedicinal, ethnobotanical, phytochemistry, and biological activities of 16 aromatic and medicinal plants from the Lamiaceae family of the Uzbek flora. Information on their traditional medicinal uses, and the compounds identified in the essential oils obtained from *Hyssopus seravschanicus*, *Leonurus panzerioides*, *L. turkestanicus*, *Mentha longifolia* var. *asiatica*, *Nepeta alatavica*, *N. olgae*, *Origanum tyttanthum*, *Perovskia scrophulariifolia*, *Phlomis thapsoides*, *Salvia korolkovii*, *S. sclarea*, *Scutellaria immaculata*, *S. ramosissima*, *S. schachristanica*, *Ziziphora clinopodioides*, and *Z. pedicellata*, has also been presented. The available literature showed that most of the bioactivities and medicinal properties of these species could be attributed to their essential oils, which contain a variety of functional bioactive compounds, known to have applications in the food, feed, pharmaceutical, and cosmetic industries.

## Figures and Tables

**Table 1:** Ethnopharmacology and biological activities of Uzbek Lamiaceae family plants.

Plant Species and Local Name	Economic Value of the Species [2]	Areal of the Plant; Collected Place; Used Part of the Plant; and Yield of Obtained Essential Oil	Traditional Usage	Documented Biological Activities of the Plant Used
*Hyssopus serawschanicus* (Dubj.) Pazij—Zarafshon issopi (Ko’ko’t)	Medicinal, essential oils containing, food (spicy flavor), honey plant	Central Asia, Iran, Afghanistan; Surkhandarya region (Khandiza); aerial parts; 0.34% [5]; 0.8%–1.0% [3]	Antiseptic, anti-inflammatory, wound healing, analgesic, antitussive and stimulating activities. Decoctions used to treat bronchial asthma, chronic bronchitis, flu and diseases of the respiratory tract. Additionally, it is used to relieve inflammation of the urinary tract [3]	The essential oil showed notable antibacterial activity against *Bacillus cereus* and *Staphylococcus aureus* [3]
*Leonurus panzerioides* Popov—Qalqonsimon bargli arslon-quyruq		Western Tien Shan, Western Pamir-Alai; Namangan region; aerial parts; 0.2% [9]	The tincture is used as a sedative and a hypotensive agent in central Asian traditional medicine [39]	Chloroform extract of this plant inhibited HL-60 cells at 48 µg/mL [39]
*L. turkestanicus* V. I. Krecz. & Kuprian—Turkiston arslon-quyrug’i	Medicinal and honey plant	Central Asia, Iran, Afghanistan, Pakistan; Tashkent region; aerial parts; 0.12% [9]	A tea and an infusion of the aboveground parts are used to treat nervous disorders, hypertension, hysteria, epilepsy, tachycardia, gastrointestinal, and female diseases, and are used as soporific, anti-inflammatory, diaphoretic, and laxative remedies. The plant is used in absent or painful menstruation, premenstrual tension, menopausal flushes. It is hypnotic and sedative and is used as a cardiac tonic [10,35,36]	Chloroform extract of this plant inhibited HL-60 cells at 26.8 µg/mL [35]
*Mentha longifolia* var. *asiatica* (Boriss) Rech. f.—Osiyo yalpizi	Medicinal, essential oils containing, food (spicy flavor), fodder, ornamental, honey plant	Central Asia, Altai; Tashkent region (Chatkal mountains); aerial parts; 0.34% [13]	An infusion and decoction of this plant is used as an anti-inflammatory, hemostatic, and is used to treat wounds, gastritis, dysenteria, diarrhea, colitis, gastralgia, tuberculosis, respiratory infections and toothaches. An infusion of the leaves and inflorescences is used as a choleretic and to treat gall bladder diseases [8]	
*Nepeta alatavica* Lipsky—Olatov nepetasi	Essential oils containing, honey plant	Tien Shan; Tashkent region; aerial parts; 0.5% [15]		The essential oil of this plant showed substantial antioxidant activity [15]
*N. olgae* Regel—Ol’ga nepetasi	Medicinal, essential oils containing, honey plant	Central Asia, Afganistan; Navoiy region; aerial parts; 1.3% [15]		The essential oil of this plant showed weak antioxidant activity [15]
*Origanum tyttanthum* Gontsch.—Mayda gulli tog’raykhon	Medicinal, essential oils containing, food, honey plant	Central Asia; Tashkent region (Chatkal mountains); aerial parts; 1.09% [18]	A decoction of the herb is used in folk medicine to stimulate the appetite and to improve digestion, to treat inflammation of mucous membranes in the upper respiratory tract, and decrease nervous excitability. Infusions and decoctions are applied externally as compresses for abscesses, and are also used in a bath to treat children who have rickets or scrofula. Water extractions of the aboveground plant parts are used to treat acute and chronic gastritis, bronchitis, cholecystitis, pneumonia, and urolithiasis and are also used as a cholagogue. A tea is used to treat tympanites, laryngitis, stomatitis, and angina, and as an oral and throat rinse [8]	It is an effective remedy to treat hypertension, atherosclerosis, kidney, liver, and epilepsy. It is a sedative for excitement of the central nervous system. A decoction of the dried leaves and flowers is used to treat intestinal atonia and as an expectorant. The plant is a component of a diaphoretic tea and is added to baths. The leaves are used as a spice and in liquor production. The essential oil has shown antimicrobial, hypocholesteremic and hypolipidemic activity [8]
		Surkhandarya region (spurs of Gissar mountains); whole plant [19]	The plant oil is used for soap-making, liquor production, fish canning and perfumery. The above ground parts of this species are used as a spice in salads, meat dishes, in salting or pickling of vegetables, in non-alcoholic (soft) drinks, and as an insecticide for dried fruit. Also in the folk medicine *O. tytthanthum* is used to treat gastritis, colitis, bronchitis, and pneumonia [19]	*O. tytthanthum* oil has been shown to possess antimicrobial effects against *Staphylococcus* sp., *Streptococcus* sp., *Pseudomonas aemginosa, Klebsiella pneumoniae* and *Salmonella* sp. [19]
*Perovskia scrophulariifolia* Bunge—Muhlisbargli perovskiya, hapri, qisroq	Medicinal, essential oils containing, dyeing, honey plant	Tien Shan, Pamir-Alai; Kashkadarya region (Yakkabag); whole plant; 0.54% [22]	The water extract is used in the bath against sun burn and applied to the skin to fight different diseases, such as dermatitis. The decoction is also consumed to fight human intestinal parasites [21]	Antibacterial activity [23]
*Phlomis thapsoides* Bunge—Sigirquyruqkabi qo’ziquloq	Essential oils containing, feeding, dyeing, honey plant	Pamir-Alai, Afghanistan; Surkhandarya region; aerial parts; 0.13% [24]	This plant is used in traditional medicine as a hemostatic and astringent drug, for treatment of wounds and stomach ache [35]	The chloroform extract inhibited HL-60 cells at IC50 = 10.6 µg/mL [35]. The EtOAc extract showed medium strength antioxidant activities. Only lamiide isolated from this plant inhibited soybean 5-LOX with IC50 value 72.92 μg/mL in vitro. The plant extracts and isolated compounds against Caco-2 and HepG-2 cancer cells indicated low cytotoxicity [24]
*Salvia korolkovii* Regel et Schmalh. (Syn. *Arischrada korolkovii*)—Korol’kov marmaragi	Essential oils containing, ornamental, honey plant	Western Tien Shan; Tashkent region (Chatkal mountains); aerial parts; 1.1% [25]	Antiseptic activity, remedy for dermatitis and cancer [26]	The essential oil and tincture prepared from roots and leaves exhibited antibacterial activity [25]. Low cytotoxic activity against P3X cells [26]
*Salvia sclarea* L.—Muskat marmarak	Medicinal, essential oils containing, food, ornamental, honey plant	South-Eastern Europe, the Mediterranean, the Caucasus, Central Asia, Iran, Afghanistan; Surkhandarya region; Inflorescence; 0.08%–1.1% [27]	The aboveground parts are used to treat fevers, stomach ulcers, headaches, epilepsy, to improve digestion, and as an antiseptic. It is used in bathes to treat bladder diseases, polyarthritis, osteomyelitis, deforming arthrosis, and trophic ulcers. The leaves are used as a antispasmodic and anti-inflammatory. A decoction of the leaves is used as a mouth washes for acute respiratory diseases and throat illnesses, periostitis and is applied externally to purulent wounds and furuncles. The decoction of the leaves and inflorescences are used to treat tachycardia and asthenia [8]	Clinical studies showed that an ointment (with 5%–20% plant extract) was highly effective in treating. An emulsion of the oil was successfully used to treat osteomylitis, varicose veins, paronychia, burns, and other diseases. In experiments, a tincture of the herb increased respiration and arterial pressure and had diuretic properties. Compounds isolated from the plant were found to be active against *Staphylococcus aureus, Candida albicans, Proteus mirabilis* [8]. Used in baths for nervous, polyarthritis, and patients with acute rheumatism [27]
*Scutellaria immaculata* Nevski ex Juz.—Dog’siz ko’kamaron		Western Tien Shan, Pamir-Alai; Namangan region; aerial parts; 0.2% [33]		Water extract of *S. immaculata* exhibited potent antioxidant activity [32]. The essential oils show moderate antioxidant activity [33]
*S. ramosissima* Popov—Sershoh ko’kamaron		Tien Shan, Northern Pamir-Alai; Tashkent region; aerial parts; 0.12% [33]	Treat epilepsy, inflammation, allergies, chorea, nervous tension, and high blood pressure [32,39]	The chloroform extract of *S. ramosissima* showed potent cytotoxic effects to cancer cells and the highest anti-trypanosomal effect against *T. b. brucei*. This extract has potent antimicrobial activity against *Streptococcus pyogenes*. Water extract of *S. ramosissima* exhibited potent antioxidant activity [32]. The essential oils show moderate antioxidant activity [33]
*S. schachristanica* Juz.—Shahriston kukamaroni		Western Pamir–Alai; Jizzakh region; aerial parts; 0.09% [33]		The essential oils show moderate antioxidant activity in DPPH, FRAP and ABTS assays [33]
*Ziziphora clinopodioides* Lam.(syn. *Ziziphora brevicalyx* Juz)—Hidli kiyiko’t	Medicinal, essential oils containing, food, honey plant	Central Asia, Western China, Siberia, Mongolia; Surkhandarya region (southwest Pamir-Alai, spur of the Hissar range, Khandiza); In inflorescences 0.6%–0.8%, in leaves 0.2%–0.3% [36]	The plant has been used since ancient times in traditional herbal medicines for the treatment of colds and cough [35]. An infusion and decoction is used to treat tachycardia, gastralgia, and heart illnesses with swelling. Juice from the plant is used as a vermifuge for pinworm in children, while an infusion of the leaves is used as an antipyretic and a decoction is used to treat typhoid fever [8]	Water and ethanol extracts of *Z. clinopodioides* showed no activity against several bacterial species, but some activity against COX-1 was recorded. The methanolic extract showed higher DPPH scavenging effect than the essential oil and other types of extracts [34]. A tincture of the herb possesses hypotensive, cardiotonic, and antihelminthic properties. The essential oil shows antibacterial and fungicidal activity. In experiments with mice, pretreatments with extracts of the plant reduced the biochemical, macro-, and microscopic effects of induced inflammatory bowel disease. Extracts and essential oil showed antibacterial activity against *Staphylococcus aureus, S. epidermidis*, *S. saprophyticus*, *Escherichia coli, Shigella flexneri*, *Salmonella typhi* and *Pseudomonas aeruginosa* [8]. *Z. clinopodioides* oil has been evaluated for insecticidal, antibacterial, antifungal and antioxidant activities [35]
*Ziziphora pedicellata* Pazij et Vved. —Gulbandli kiyiko’t	Medicinal, essential oils containing, food, honey plant	Tien Shan; Tashkent region (Chimgan area); aerial parts; 0.53% [38]	Used in cases of gastric intestinal and cardiovascular diseases [38]. A tincture and decoction of the aboveground parts are used as a diuretic and the fresh ground plant is used to heal wounds. An infusion of the herb, taken as a tea, is used as a hypotensive and to treat headaches [40]	Dry extract showed restoring impact on the functional condition of liver at its damage by alcohol and can be recommended as pathogenetic preparation for the treatment of diseases of hepatobiliary system [37]. In pharmacological studies, infusions, tinctures and liquid extracts of this plant had positive effects on myocarditis and myocardial infarction. The same preparations acted as a cardiotonic, decreased arterial pressure, and increased diuresis [40]

## References

[1] Tojibaev K., Beshko N., Turginov O., Mirzalieva D. (2014). New records for Fabaceae in the flora of Uzbekistan. Fl. Medit..

[2] Virtual Guide to the Flora of Uzbekistan Plant Database as Practical Approach. http://floruz.uz/plants.

[3] Sharopov F.S., Kukaniev M.A., Thompson R.M., Satyal P., Setzer W.N. (2012). Composition and antimicrobial activity of the essential oil of *Hyssopus seravschanicus* growing wild in Tajikistan. Der Pharm. Chem..

[4] Zotov E.P., Goryaev M.I., Sharipova F.S., Khazanovich R.L., Vandysheva V.I. (1974). Investigation of the essential oil of *Hyssopus zeravshanicus*. Chem. Nat. Comp..

[5] Dzhumaev K.H.K., Zenkevich I.G., Tkachenko K.G., Tsiburskaya I.A. (1990). Essential oil of the leaves of *Hyssopus seravschanicus* from South Uzbekistan. Chem. Nat. Comp..

[6] Huang Z., Zhu Z.X., Li Y.T., Pang D.R., Zheng J., Zhang Q., Zhao Y.F., Ferreira D., Zjawiony J.K., Tu P.F. (2015). Anti-inflammatory labdane diterpenoids from *Leonurus macranthus*. J. Nat. Prod..

[7] Vvedenskiy A. (1961). Flora Uzbekistana (Flora of Uzbekistan).

[8] Eisenman S.W., Zaurov D.E., Struwe L. (2013). Medicinal Plants of Central Asia: Uzbekistan and Kyrgyzstan.

[9] Mamadalieva N.Z., Bobakulov K.M., Vinciguerra V., Tiezzi A., Abdullaev N.D., Nahar L., Azimova S.S., Sarker S.D. (2016). GC-MS and q-NMR based chemotaxonomic evaluation of two *Leonurus* species. Phytochem. Anal..

[10] Mamedov N., Mamadalieva N. (2016). Medicinal Plants from Countries of Former USSR Used for Treatment of Depression. Herbal Medicine in Depression: Traditional Medicine to Innovative Drug Delivery.

[11] Khalmatov K.K. (1964). Dikorastushchiye Lekarstvenniye Rasteniya Uzbekistana (Wild-Growing Medicinal Plants of Uzbekistan).

[12] Kumar P., Mishra S., Malik A., Satya S. (2011). Insecticidal properties of *Mentha* species: A review. Ind. Crops Prod..

[13] Baser K.H.C., Nuriddinov K.H.R., Nigmatullaev A.M., Aripov K.H.N. (1997). Essential oil of *Mentha asiatica* Boriss. from Uzbekistan. J. Essent. Oil Res..

[14] Formisano C., Rigano D., Senatore F. (2011). Chemical constituents and biological activities of *Nepeta* species. Chem. Biodiv..

[15] Mamadalieva N.Z., Sharopov F.S., Satyal P., Azimova S.S., Wink M. (2016). Analysis of the chemical composition of the essential oils of some Central Asian *Nepeta* species (Lamiaceae) by GLC-MS. Nat. Prod. Commun..

[16] Tepe B., Cakir A., Sihoglu Tepe A. (2016). Medicinal uses, phytochemistry, and pharmacology of *Origanum onites* (L.): A Review. Chem. Biodiv..

[17] Takeda Y., Tomonari M., Arimoto S., Masuda T., Otsuka H., Matsunami K., Honda G., Ito M., Takaishi Y., Kiuchi F. (2008). A new phenolic glucoside from an Uzbek medicinal plant, *Origanum tyttanthum*. J. Nat. Med..

[18] Baser K.H.C., Demirçakmak B., Nuriddinov K.H.R., Nigmatullaev A.M., Aripov K.H.N. (1997). Composition of the essential oil of *Origanum tyttanthum* Gontsch. from Uzbekistan. J. Essent. Oil Res..

[19] Dzumayev K.H.K., Tkachenko K.G., Zenkevich I.G., Tsibulskaya I.A. (1999). Essential oils of *Origanum tytthanthum* Gontsch. produced from plants grown in Southern Uzbekistan. J. Essent. Oil Res..

[20] Perveen S., Malik A., Tareen R.B. (2009). Phytochemical studies on *Perovskia atriplicifolia*. J. Chem. Soc. Pak..

[21] Takeda Y., Hayashi T., Masuda T., Honda G., Takaishi Y., Ito M., Otsuka H., Matsunami K., Khodzhimatov O.K., Ashurmetov O.A. (2007). Chemical constituents of an Uzbek medicinal plant, *Perovskia scrophularifolia*. J. Nat. Med..

[22] Nuriddinov K.R., Khodzimatov K.K., Aripov K.N., Ozek T., Demirchakmak B., Basher K.H.C. (1997). Essential oil of *Perovskia scrophularifolia*. Chem. Nat. Comp..

[23] Abduganiev B.E., Abdullaev U.A., Plugar V.N. (1995). Qualitative and quantitative compositions of the essential oil of *Perovskia scrophulariifolia*. Chem. Nat. Comp..

[24] Sobeh M., Mamadalieva N.Z., Mahmoud T., Krstin S., Youssef F.S., Ashour M.L., Azimova S.S., Wink M. (2016). Chemical profiling of *Phlomis thapsoides* (Lamiaceae) and in vitro testings of biological activities. Med. Chem. Res..

[25] Mamadalieva N.Z., Egamberdieva D., Climati E., Triggiani D., Ceccarelli D., Sultanov S.S., Nigmatullaev A.M., Azimova S.H.S., Tiezzi A. (2009). The cytotoxic activities of four *Salvia* species native for Uzbekistan. Uzbek Biol. J..

[26] Baser K.H.C., Nuriddinov H.R., Ozek T., Demirci B., Azcan N., Nigmatullaev A.M. (2002). Essential oil of *Arischrada korolkowii* from the Chatkal mountains of Uzbekistan. Chem. Nat. Comp..

[27] Dzumayev K.H.K., Tsibulskaya I.A., Zenkevich I.G., Tkachenko K.G., Satzyperova I.F. (1995). Essential oils of *Salvia sclarea* L. produced from plants grown in Southern Uzbekistan. J. Essent. Oil Res..

[28] Kuźma Ł., Kalemba D., Różalski M., Różalska F., Więckowska-Szakiel M., Krajewska U., Wysokińska H. (2009). Chemical composition and biological activities of essential oil from *Salvia sclarea* plants regenerated in vitro. Molecules.

[29] Pitarokili D., Couladis M., Petsikos-Panayotarou N., Tzakou O. (2002). Composition and antifungal activity on soil-borne pathogens of the essential oil of *Salvia sclarea* from Greece. J. Agric. Food Chem..

[30] Sharopov F.S., Setzer W.N. (2012). The Essential oil of *Salvia sclarea* L. from Tajikistan. Rec. Nat. Prod..

[31] Shang X., He X., He X., Li M., Zhang R., Fan P., Zhang Q., Jia Z. (2010). The genus *Scutellaria* an ethnopharmacological and phytochemical review. J. Ethnopharmacol..

[32] Mamadalieva N.Z., Herrmann F., El-Readi M.Z., Tahrani A., Hamoud R., Egamberdieva D., Azimova S.S., Wink M. (2011). Flavonoids in *Scutellaria immaculata* and *S. ramosissima* (Lamiaceae) and their biological activity. J. Pharm. Pharmacol..

[33] Mamadalieva N.Z., Sharopov F.S., Satyal P., Azimova S.S., Wink M. (2016). Composition of the essential oils of three Uzbek *Scutellaria* species (Lamiaceae) and their antioxidant activities. Nat. Prod. Res..

[34] Šmejkal K., Malaník M., Zhaparkulova K., Sakipova Z., Ibragimova L., Ibadullaeva G., Žemliˇcka M. (2016). Kazakh *Ziziphora* species as sources of bioactive substances. Molecules.

[35] Sharopov F.S., Setzer W.N. (2011). Chemical diversity of *Ziziphora clinopodioides*: Composition of the essential oil of *Z. clinopodioides* from Tajikistan. Nat. Prod. Commun..

[36] Dzhumaev K.H.K., Zenkevich I.G., Tkachenko K.G., Tsibui'skaya I.A. (1990). Essential oils of the inflorescences and leaves of *Ziziphora brevicalyx*. Chem. Nat. Compd..

[37] Mavlanov S.H.R., Khakimov Z.Z., Rakhmanov A.K.H., Khodzimatov O.K. (2015). The importance of dry extract from plants of Central Asia for eliminating alterations of functional state of liver in its alcoholic lesion. Am. J. Med. Med. Sci..

[38] Dembitskii A.D., Bergaliev E.S.H., Kyazimov I.M. (1994). Chemical composition of the essential oils *Ziziphora* growing under various ecological conditions. Chem. Nat. Compd..

[39] Mamadalieva N.Z., Mamedov N.A., Craker L.E., Tiezzi A. (2014). Ethnobotanical uses and cytotoxic activities of native plants from the Lamiaceae family in Uzbekistan. Acta Hort..

[40] Egamberdieva D., Mamadalieva N., Khodjimatov O., Tiezzi A. (2013). Medicinal plants from Chatkal Biosphere Reserve used for folk medicine in Uzbekistan. Med. Arom. Plant Sci. Biotechnol..

